# Clinical performance of the VITEK REVEAL fast antimicrobial susceptibility test within a real-world workflow for gram-negative bacteremia: comparison with QMAC-dRAST and conventional methods

**DOI:** 10.1128/spectrum.01972-25

**Published:** 2025-11-28

**Authors:** Junghun Park, Hae-Sun Chung, Mihwa Kim, Yohann Bala, Geehay Hong, Miae Lee, Min-Kyung So

**Affiliations:** 1 Ewha Medical Research Institute, Ewha Womans University College of Medicine 92203, Seoul, Republic of Korea; 2 Department of Laboratory Medicine, Ewha Womans University College of Medicine 92203, Seoul, Republic of Korea; 3 Global Medical Affairs, bioMérieux, Marcy L'Etoile, France; 4 Medical Affairs, bioMérieux Korea, Seoul, Republic of Korea; 5 Department of Laboratory Medicine, Seegene Medical Foundation 595499 , Seoul, Republic of Korea; Indiana University School of Medicine, Indianapolis, Indiana, USA

**Keywords:** bloodstream infections, gram-negative bacteria, fast antimicrobial susceptibility testing, time-to-result, volatile organic compounds, phenotypic resistance, genotypic resistance, blood culture systems

## Abstract

Fast antimicrobial susceptibility testing (AST) is essential for appropriate antimicrobial therapeutic guidance in patients with bloodstream infections, reducing the time required for optimal treatment. We aimed to evaluate the clinical performance of the VITEK REVEAL (REVEAL), a volatile organic compound-based fast AST system, compared with our already established AST workflow for bloodstream infections. We prospectively collected a total of 107 gram-negative isolates from positive blood cultures and performed rapid species identification using the BIOFIRE BCID2 panel, followed by AST using the REVEAL system. We conducted parallel comparative tests (QMAC-dRAST, VITEK 2, and ETEST for selected antimicrobials) and analyzed the results for categorical agreement (CA), essential agreement (EA), and categorical discrepancies. Moreover, we compared the time to results between the REVEAL vs QMAC-dRAST and VITEK2. Compared with QMAC-dRAST, REVEAL demonstrated 94.1% CA and 98.4% EA across 1,200 pairwise comparisons, with very major discrepancy (VMD) and major discrepancy (MD) rates of 1.4% and 0.7%, respectively. Compared to VITEK2 (872 comparisons), REVEAL yielded 94.5% CA and 97.9% EA, with VMD and MD of 0.5% and 0.6%, respectively. For ceftazidime/avibactam and ceftolozane/tazobactam, REVEAL showed 100% and 98.6% CA, respectively, compared to ETEST. The median final and per-antibiotic time to results for REVEAL were 8.00 h (range: 6.50–8.15 h) and 5.97 h (range: 3.17–8.15 h), respectively. The REVEAL system demonstrated high concordance with existing fast and conventional AST methods and enabled reliable susceptibility testing. These results support the potential integration of the REVEAL system into routine microbiological workflows for time-sensitive clinical decision-making in bloodstream infections.

**IMPORTANCE:**

Fast antimicrobial susceptibility testing is critical for initiating appropriate therapy in patients with gram-negative bacteria-induced bloodstream infections. Conventional antimicrobial susceptibility testing methods often require extended turnaround times, thereby delaying optimal treatment. In this study, we prospectively evaluated the VITEK REVEAL system, which detects bacterial growth-associated volatile organic compounds to directly determine antimicrobial susceptibility in positive blood cultures. We assessed the system against our already established identification and antimicrobial susceptibility testing methods, including both fast and conventional platforms currently implemented in routine clinical practice. The volatile organic compounds-based system demonstrated high concordance, shorter time-to-results, and reliable performance for newer antimicrobial agents. By directly comparing its performance with established clinical diagnostic tools, this study provides relevant support for the potential integration of this approach into real-world laboratory workflows, with implications for improved patient management and antimicrobial stewardship.

## INTRODUCTION

Despite advances in antimicrobial therapy, bloodstream infections (BSIs) pose a constant challenge due to their high mortality rates (1). Gram-negative bacteria-induced BSIs are of particular concern due to their rapid progression and frequent association with antimicrobial resistance, complicating the selection of effective therapies (2, 3).

Early antimicrobial susceptibility testing (AST) is essential to initiate timely and appropriate antimicrobial treatment (4). However, conventional AST methods typically require isolate subculturing and at least 48 h to deliver results for result delivery following a positive blood culture, thereby delaying optimal therapeutic decisions. To address this gap, various fast AST platforms have been developed and adopted recently. Systems such as dRAST (QMAC-dRAST, Quantamatrix, South Korea) and Accelerate Pheno system (Accelerate Diagnostics, USA) enable direct AST from positive blood culture bottles within several hours, and their clinical utility has been increasingly recognized (5–8). Rapid AST implementation not only significantly reduces result generation time but also improves antimicrobial therapy, considered to potentially improve patient outcomes (9).

The VITEK REVEAL (REVEAL, bioMérieux Inc., Durham, NC, USA) system, granted FDA 510(k) clearance in June 2024, introduces a novel approach to rapid AST by integrating metabolomic profiling and microsensor technology to detect bacterial growth-indicating volatile organic compounds (VOC) (10, 11). It directly determines in real time the minimum inhibitory concentration (MIC) in positive blood culture specimens. Notably, the system provides susceptibility results for new antimicrobial agents (e.g., ceftazidime/avibactam and ceftolozane/tazobactam), enabling earlier-initiation of targeted therapy for multidrug-resistant gram-negative infections.

In this study, we aimed to evaluate the clinical AST performance of the REVEAL system, on gram-negative bacteria directly from positive blood culture. We compared REVEAL with both fast (QMAC-dRAST) and conventional (VITEK2, and ETEST) AST methods currently used in our clinical practice. We assessed the concordance, Time-to-Result (TTR), and potential clinical utility of the REVEAL in a real-world diagnostic setting. To the best of our knowledge, this represents one of the first prospective real-world VOC-based fast AST technology evaluations within the above routine diagnostic workflows, and the first to do so in accordance with CLSI M100 breakpoints.

## MATERIALS AND METHODS

### Bacterial isolates and study design

We prospectively collected a total of 107 consecutive gram-negative isolates from positive blood cultures at Ewha Womans University Mokdong Hospital, a tertiary care center, between December 2023 and October 2024. The study protocol was approved by the Institutional Review Board (IRB No. 2023-05-014-002) and conducted in accordance with the Declaration of Helsinki and Good Clinical Practice guidelines.

The patient population was primarily from the Intensive Care Unit. We subjected blood cultures flagged as positive to Gram staining and included only gram-negative organisms in this study. Positive blood cultures were processed on the BACT/ALERT VIRTUO system (bioMérieux, Marcy-l’Étoile, France), and aliquots from BACT/ALERT FA Plus, and FN Plus bottles were used in this study. The isolates collected comprised *Enterobacterales* and non-fermenting gram-negative bacilli recovered from positive blood cultures during the study period (see Table 1 for species distribution). When multiple blood culture bottles from the same patient were tested positive, the earliest flagged bottle was used for testing. We excluded specimens identified as polymicrobial by BCID2 from the study. To ensure analytical accuracy and in accordance with the manufacturer’s Instructions for Use, we excluded samples from REVEAL testing if over 16 h had passed after blood culture positivity, as delayed processing might induce metabolic changes affecting VOC-based susceptibility profiling.

**TABLE 1:** Distribution of species and detection rates of resistance genes^*a*^

Species	Enrolled no.	Anti-microbial resistance gene detection in BCID2, % (No.)
*Escherichia coli*	64	CTX-M 29.7% (20), CTX-M and KPC 3.1% (2)
*Klebsiella pneumoniae* group	21	CTX-M 14.3% (3), CTX-M and KPC 9.5% (2)
*Pseudomonas aeruginosa*	6	NDM 33.3% (2)
*Acinetobacter baumannii* complex/group	2	ND
*Klebsiella oxytoca*	2	ND
*Enterobacter cloacae* complex	2	CTX-M and NDM 50% (1)
*Klebsiella aerogenes*	1	ND
*Proteus mirabilis*	1	CTX-M 100% (1)
*Proteus vulgaris*	1	ND
Overall	100	

^*a*^ CTX-M, cefotaximase-Munich-type extended-spectrum β-lactamase; KPC, Klebsiella pneumoniae carbapenemase; NDM, New Delhi metallo-β-lactamase; ND, not detected; BCID2, BioFire Blood Culture Identification 2 Panel.

### Fast ID/AST vs conventional ID/AST workflows

**Fig 1:**
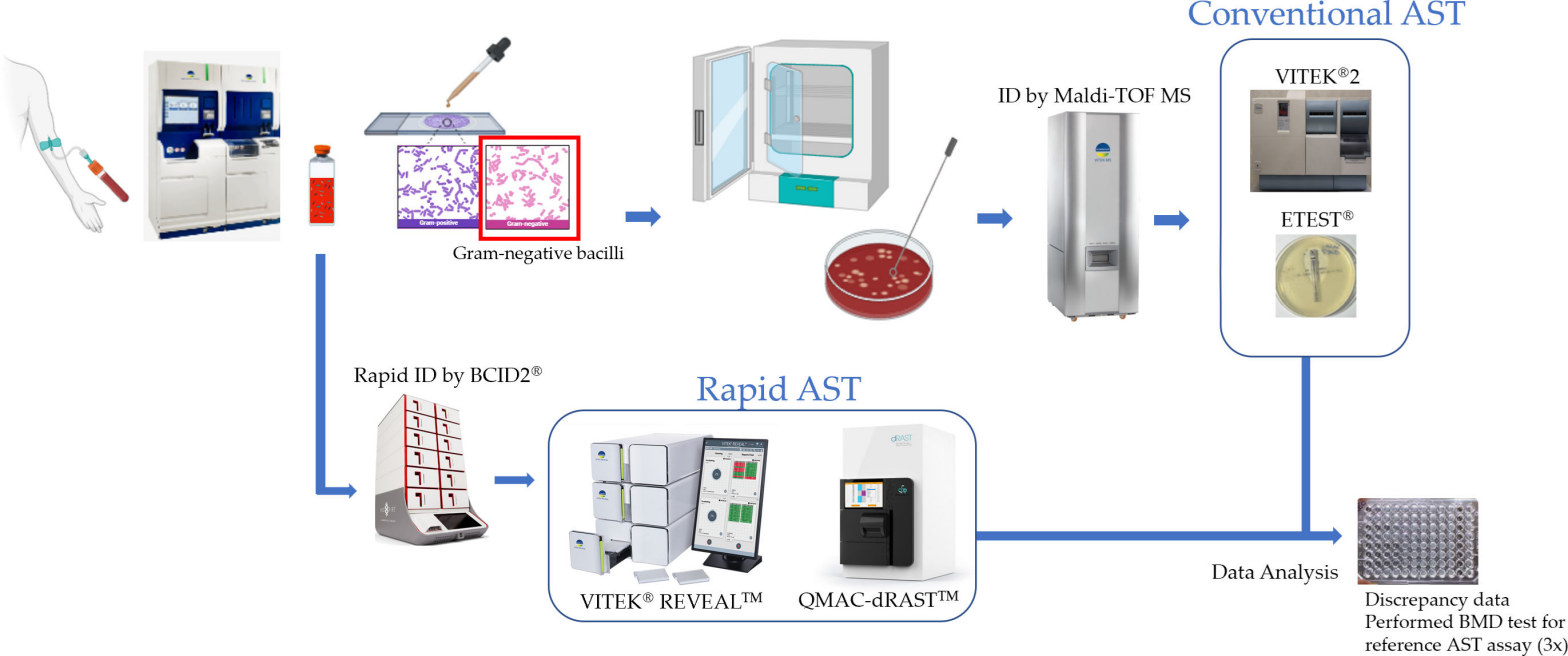
Overview of the study workflow: The study procedures followed standard clinical protocols, with the REVEAL (VITEK REVEAL) system incorporated for comparative evaluation. After the rapid identification of organisms using the BIOFIRE BCID2 panel, rapid AST was conducted simultaneously using our already established QMAC-dRAST system and the REVEAL system. The REVEAL results were also compared with conventional AST data obtained using VITEK2 or, for selected antibiotics, the ETEST. Discrepant results were confirmed using broth microdilution as the reference method. QuantaMatrix Inc. hereby grants permission to use the image of the dRASTTM system in Figure 1 of the manuscript to be published in Microbiology Spectrum.

We followed an internal protocol for implementing fast identification (ID) and fast AST workflow (Fig. 1). We performed species ID using the BIOFIRE BCID2 panel (bioMérieux, Salt Lake City, UT, USA).

Each positive blood culture sample underwent direct AST using both the REVEAL (GN BC-AST RUO panel) and QMAC-dRAST (GN S17 panel, QuantaMatrix, South Korea; software version: 1.5.1.016) systems according to the manufacturer’s instructions.

In parallel, we performed conventional AST workflows, including subculturing onto blood agar, colony ID by matrix assisted laser desorption ionization–time of flight mass spectrometry (MALDI–TOF–MS) using VITEK MS (bioMérieux, Marcy-L’Etoile, France), and AST using the VITEK2 system (bioMérieux Inc., Durham, NC, USA, AST-GN cards N413 for *Enterobacterales*/N414 for glucose non-fermenting Gram-negative bacilli). Because ceftazidime/avibactam and ceftolozane/tazobactam are not included in the VITEK 2 AST-GN N413/N414 or QMAC-dRAST GN S17 panels, susceptibility to these agents was determined using ETEST (bioMérieux, Marcy-l’Étoile, France) in accordance with standard guidelines.

### Antimicrobial selection for comparative assessment

Comparative testing focused on antimicrobials approved under FDA 510(k) clearance for REVEAL, with intended use limitations applied as appropriate (10). Table S1 presents the list of the antimicrobial agents tested for each system. The number of paired results compared may vary by antimicrobial agent depending on each system’s testing limitations and regulatory claims. Detailed information is provided in Table S1.

### Interpretation criteria and performance metrics

We interpreted the AST results based on the Clinical and Laboratory Standards Institute (CLSI) M100 (34th edition, 2024) MIC breakpoints for each organism-antimicrobial combination (12). ESBL was evaluated only for *Enterobacterales* species (*E. coli*, *K. pneumoniae*, and *K. oxytoca*). For each isolate, ESBL-positive or -negative results were recorded as reported by the automated AST systems (REVEAL, QMAC-dRAST, and VITEK 2). In the REVEAL system, ESBL determination was based on MICs of cefotaxime and ceftazidime, both alone and in combination with clavulanate. We defined the agreement and discrepancy metrics between REVEAL and the comparator systems (QMAC-dRAST, VITEK2, or ETEST) as follows:

Categorical Agreement (CA): matching susceptibility category proportions, susceptible (S), intermediate (I), and resistant (R), between REVEAL and comparative methods.

Essential Agreement (EA): MIC result proportions within ±1 twofold dilution between the methods.

Very Major Discrepancy (VMD): comparator result = resistant; REVEAL = susceptible.

Major Discrepancy (MD): comparator result = susceptible; REVEAL = resistant.

Minor Discrepancy (miD): Discrepancies involving the intermediate category or S vs I or R vs I discrepancies.

### Reference testing with broth microdilution

For all VMD or MD cases, broth microdilution (BMD) testing was performed in triplicate by an independent Reference laboratory according to Clinical and Laboratory Standards Institute guidelines (CLSI) (13). BMD testing panels were manufactured by JMI Laboratories according to their specifications, in compliance with the CLSI M07 standard, and stored frozen until testing. We considered the modal MIC value from three replicates the reference standard for the isolate-antibiotic combination.

### TTR analysis

We defined TTR as the time elapsed from the start of sample loading to final result reporting. In addition, we recorded per-antibiotic TTR values for REVEAL to characterize the real-time reporting functionality. TTR comparisons were performed using the two-sided Mann-Whitney *U* test in MedCalc Version 20.011 (MedCalc Software Ltd., Ostend, Belgium); *P* < 0.05 was considered statistically significant.

## RESULTS

### Bacterial isolate distribution

Of the 107 initially collected positive blood culture samples, 100 isolates were included in the final analyses, and 7 samples were excluded due to delayed REVEAL loading beyond 16 h (*n* = 4), reagent failure (*n* = 1), and mixed colonies on subculture (*n* = 2). One additional isolate failed to generate QMAC-dRAST results and was excluded only from the REVEAL vs QMAC-dRAST comparison. The dominant species were *E. coli* (*n* = 64), *K. pneumoniae* group (*n* = 21), other *Enterobacterales* (*n* = 7), *P. aeruginosa* (*n* = 6), and *A. baumannii* complex/group (*n* = 2) (Table 1). No isolates of *Citrobacter freundii* complex, *Citrobacter koseri*, or *Serratia marcescens* were recovered during the study period. Analysis of antimicrobial resistance (AMR) genes detected by the BCID2 panel identified CTX-M genes in 29 isolates, among which five also carried KPC or NDM DNA profile. Two *P. aeruginosa* isolates harbored NDM only. A total of 69 isolates were negative for all the tested resistance genes.

### Comparison of REVEAL and comparator methods

A total of 1,200 REVEAL–QMAC-dRAST antimicrobial-organism pairs were analyzed, and 19.3% (232/1200) of them were classified as resistant from the REVEAL testing. Among the 1,200 pairs, the overall CA and EA were 94.1% and 98.4%, respectively (Table 2). The VMD and MD rates were 1.4% (3/219) and 0.7% (6/900), respectively. The ESBL-CA of the system was 100%. However, six isolates (three *E. coli* and three *K. pneumoniae*) were excluded from the analysis: one isolate yielded indeterminate results in both REVEAL and QMAC-dRAST, and five isolates yielded indeterminate results in QMAC-dRAST. The CA was below 90% for Ampicillin/Sulbactam and Aztreonam. However, their EA rates were 98.5% and 96.8%, respectively, indicating that most discrepancies were minor.

**TABLE 2:** Comparison of antimicrobial susceptibility testing results between REVEAL and QMAC-dRAST systems^*a*^^,^^*b*^^,^^*c*^

Antimicrobial	n.Pwc	REVEAL			QMAC-dRAST			Agreement						
		S	I	R	S	I	R	n.CA	%CA	n.EA	%EA	n.VMD	n.MD	n.miD
Amikacin	91	85	4	2	89	0	2	87	95.6	91	100.0	0	0	4
Amoxicillin/clavulanate	80	61	4	15	58	7	15	75	93.8	80	100.0	0	0	5
Ampicillin/sulbactam	66	29	19	18	23	26	17	54	81.8	65	98.5	0	1	11
Aztreonam	94	67	2	25	63	9	22	84	89.4	91	96.8	1	0	9
Cefepime	95	66	6	23	67	11	17	87	91.6	90	94.7	0	1	7
Cefotaxime	91	58	0	33	54	4	33	87	95.6	90	98.9	0	0	4
Ceftazidime	91	72	0	19	63	8	20	82	90.1	90	98.9	1	0	8
Ciprofloxacin	96	48	7	41	49	6	41	87	90.6	93	96.9	0	2	7
Ertapenem	86	80	3	3	82	1	3	84	97.7	86	100.0	0	0	2
Gentamicin	88	64	0	24	64	0	24	88	100.0	88	100.0	0	0	0
Imipenem	94	86	1	7	86	2	6	92	97.9	92	97.9	0	1	1
Meropenem	96	89	0	7	89	1	6	95	99.0	96	100.0	0	0	1
Piperacillin/tazobactam	83	70	6	7	72	7	4	79	95.2	81	97.6	0	1	3
Trimethoprim/sulfamethoxazole	49	41	0	8	40	0	9	48	98.0	48	98.0	1	0	0
Overall	1,200	917	52	232	900	82	219	1,129	94.1	1,181	98.4	3	6	62

^*a*^ CA, categorical agreement; EA, essential agreement; I, intermediate; n.CA, number of categorical agreements; n.EA, number of essential agreements; n.MD, number of major discrepancies; n.miD, number of minor discrepancies; n.VMD, number of very major discrepancies; n.Pwc, number of pairwise comparisons; R, resistant; S, susceptible; REVEAL, VITEK REVEAL.

^*b*^ Sample No. 99; QMAC-dRAST results could not be obtained for one sample due to data failure.

^*c*^ The categorical agreement for extended-spectrum β-lactamase (ESBL) detection between REVEAL and QMAC-dRAST was 100% (80/80; 23 positive and 57 negative isolates). Six Pwc were excluded due to indeterminate interpretations (REVEAL and QMAC-dRAST, *n* = 1; QMAC-dRAST only, *n* = 5).

In the 872 REVEAL–VITEK2 pairs, CA and EA were 94.5% and 97.9%, respectively (Table 3). The observed VMD and MD rates were 0.5% (1/195) and 0.6% (4/648), respectively. The CA for ESBL detection was 96.5%. The discrepant isolates included one *E. coli* and two *K. pneumoniae*. The CA was <90% for Ceftazidime and Ciprofloxacin, mostly due to minor discrepancies (11 and 9, respectively), although the EA remained high (i.e., 98.9% and 99.0%, respectively).

**TABLE 3:** Comparison of antimicrobial susceptibility testing results between REVEAL and VITEK2 systems^*a*^^,^^*b*^

Antimicrobial	n.Pwc	REVEAL			VITEK2			Agreement						
		S	I	R	S	I	R	n.CA	%CA	n.EA	%EA	n.VMD	n.MD	n.miD
Amoxicillin/clavulanate	80	61	4	15	58	5	17	75	93.8	80	100.0	0	0	5
Cefepime	90	62	7	21	65	2	23	81	90.0	85	94.4	0	0	9
Cefotaxime	92	58	0	34	58	0	34	92	100.0	92	100.0	0	0	0
Ceftazidime	92	73	0	19	64	11	17	80	87.0	91	98.9	1	0	11
Ciprofloxacin	97	48	8	41	44	9	44	87	89.7	96	99.0	0	1	9
Ertapenem	85	80	2	3	81	1	3	82	96.5	84	98.8	0	0	3
Gentamicin	87	63	0	24	64	0	23	86	98.9	86	98.9	0	1	0
Imipenem	95	87	1	7	87	0	8	94	98.9	94	98.9	0	0	1
Levofloxacin	6	3	0	3	3	0	3	6	100.0	6	100.0	0	0	0
Meropenem	8	4	0	4	4	0	4	8	100.0	8	100.0	0	0	0
Piperacillin/tazobactam	84	71	6	7	73	1	10	78	92.9	77	91.7	0	1	5
Tobramycin	6	4	0	2	4	0	2	6	100.0	6	100.0	0	0	0
Trimethoprim/sulfamethoxazole	50	42	0	8	43	0	7	49	98.0	49	98.0	0	1	0
Overall	872	656	28	188	648	29	195	824	94.5	854	97.9	1	4	43

^*a*^ CA, categorical agreement; EA, essential agreement; ESBL, extended-spectrum β-lactamase; I, intermediate; n.CA, number of categorical agreements; n.EA, number of essential agreements; n.MD, number of major discrepancies; n.miD, number of minor discrepancies; n.VMD, number of very major discrepancies; n.Pwc, number of pairwise comparisons; R, resistant; S, susceptible; REVEAL, VITEK REVEAL.

^*b*^ The categorical agreement for extended-spectrum β-lactamase (ESBL) detection between REVEAL and VITEK2 was 96.5% (83/86; REVEAL, 28 positive and 58 negative isolates; VITEK2, 27 positive and 59 negative isolates). One Pwc was excluded due to an indeterminate interpretation by REVEAL.

A comparison of REVEAL and ETEST for ceftazidime/avibactam and ceftolozane/tazobactam revealed CA values of 100% and 98.6%, respectively (Table 4). We observed no significant discrepancies. One miD occurred for ceftolozane/tazobactam.

**TABLE 4:** Comparison of ceftazidime/avibactam and ceftolozane/tazobactam susceptibility testing between REVEAL and ETEST^*a*^

Antimicrobial	n.Pwc	REVEAL			ETEST			Agreement						
		S	I	R	S	I	R	n.CA	%CA	n.EA	%EA	n.VMD	n.MD	n.miD
Ceftazidime/avibactam	95	92	0	3	92	0	3	95	100.0	94	98.9	0.0	0	0
Ceftolozane/tazobactam	74	70	0	4	69	1	4	73	98.6	74	100.0	0.0	0	1
Overall	169	162	0	7	161	1	7	168	99.4	168	99.4	0.0	0	1

^*a*^ CA, categorical agreement; EA, essential agreement; I, intermediate; n.CA, number of categorical agreements; n.EA, number of essential agreements; n.MD, number of major discrepancies; n.miD, number of minor discrepancies; n.VMD, number of very major discrepancies; n.Pwc, number of pairwise comparisons; R, resistant; S, susceptible; REVEAL, VITEK REVEAL.

The original raw data corresponding to these analyses were provided in Tables S2 to S5 for detailed reference.

### Discrepancy resolution with broth microdilution

Twelve drug-bug combinations exhibiting VMD or MD results from REVEAL vs QMAC-dRAST or VITEK2 were subjected to BMD testing in triplicate (Table 5). BMD interpretation was excluded for one pair due to the absence of modal MIC. Among the remaining 11 pairs, 7 (63.6%) displayed categorical agreement with REVEAL, whereas we classified 4 pairs as REVEAL errors: one VME (*E. coli*–aztreonam) and three MEs (*E. coli*–Piperacillin/Tazobactam; *E. coli*–Ampicillin/Sulbactam; *K. pneumoniae*–ciprofloxacin).

**TABLE 5:** Analysis of very major and major discrepancies: comparison of MICs across REVEAL, QMAC-dRAST, and VITEK2 using broth microdilution as the reference standard^*a*^

Species	Antimicrobial	REVEAL MIC	REVEAL interpretation	QMAC-dRAST MIC	QMAC-dRAST interpretation	VITEK2 MIC	VITEK2 interpretation	BMD (3×) MIC	BMD interpretation	Determination of results
Very major discrepancies										
*E. coli*	Trimethoprim/sulfamethoxazole	≤2	S	≥8	R	≤1	S	0.12, 0.12, 0.12	S	Agreement
*E. coli*	Aztreonam	≤4	S	≥32	R	NA	NA	>32, >32, >32	R	VME
*E. coli*	Ceftazidime	≤4	S	16	R	8	I	8, 16, 32	R^*b*^	Inconclusive
*E. coli*	Ceftazidime	≤4	S	8	I	32	R	4, 8, 4	S	Agreement
Major Discrepancies										
*E. coli*	Imipenem	4	R	≤1	S	8	R	4, 4, 4	R	Agreement
*E. coli*	Cefepime	16	R	2	S	16	R	32, 32, 32	R	Agreement
*E. coli*	Ciprofloxacin	>2	R	0.25	S	≥4	R	>8, >8, >8	R	Agreement
*K. pneumoniae*	Ciprofloxacin	1	R	0.25	S	0.12	S	0.12, 0.12, 0.12	S	ME
*E. coli*	Piperacillin/tazobactam	64	R	≤8	S	≤4	S	2, 2, 2	S	ME
*E. coli*	Ampicillin/sulbactam	>16	R	≤8	S	NA	NA	8, 8, 8	S	ME
*E. coli*	Gentamicin	>8	R	≥16	R	≤1	S	>64, >64, >64	R	Agreement
pneumoniae	Trimethoprim/sulfamethoxazole	>4	R	4	R	≤1	S	>16, >16, >16	R	Agreement

^*a*^ BMD, broth microdilution; MIC, minimum inhibitory concentration; R, resistant; I, intermediate; S, susceptible; NA, not available; VME, very major error; ME, major error; REVEAL, VITEK REVEAL.

^*b*^ BMD failed to conclude on modal MIC.

### TTR comparison

The median final TTR for the REVEAL system was 8.00 h (6.50–8.15), this was statistically significantly longer (*P* < 0.0001) than that for QMAC-dRAST (median: 6.63 h [4.48–8.13]) (Table 6). This longer final TTR for REVEAL was driven by the longest drug to be released, particularly for meropenem and piperacillin/tazobactam (Fig. S1). REVEAL allowing a release of MIC and interpreted categories on a drug per drug basis, the median per antibiotic TTR for REVEAL was 5.97 h (3.17—8.15). Compared to the conventional VITEK2 workflow (median: 9.45 h [6.16—18.00], excluding subculture time), REVEAL provided results earlier.

**TABLE 6:** Comparison of time to result across antimicrobial susceptibility testing platforms^*a*^

Technique	TTR measurement interval	Median (h)	Min (h)	Max (h)
REVEAL	Loading to final results	8.00	6.50	8.15
	Loading to individual antibiotic release	5.97	3.17	8.15
QMAC-dRAST	Loading to final results	6.63	4.48	8.13
VITEK2^*b*^	Loading to final results	9.45	6.17	18.00

^*a*^ TTR, time-to-result; REVEAL, VITEK REVEAL.

^*b*^ From incubation to results, this does not account for time to get isolated colonies from positive blood culture.

### Correlation between genotype and phenotypic susceptibility

Among the 100 isolates tested, 31 were positive for resistance genes (CTX-M, KPC, or NDM) according to the BCID2 panel. Of these, 29 carried the CTX-M gene, including 27 *E. coli* or *K. pneumoniae* isolates, 1 *E. cloacae* complex, and 1 *Proteus mirabilis* isolate (Table 7). According to the REVEAL system, all 28 *E. coli*, *K. pneumoniae,* and *E. cloacae* complex isolates were resistant to cefotaxime, while 14 strains were susceptible to ceftazidime.

**TABLE 7:** Correlation between genotypic resistance detected by BCID2 and phenotypic antimicrobial susceptibility by REVEAL^*a*^^,*b*^

Antibiotic resistance gene detection by BCID2		REVEAL cefotaxime				REVEAL ceftazidime				REVEAL ESBL				REVEAL imipenem				REVEAL meropenem				REVEAL ertapenem			
Organism	n	R	I	S	NA	R	I	S	NA	+	−	Ind	NA	R	I	S	NA	R	I	S	NA	R	I	S	NA
CTX-M	24	23			1	10		13	1	23			1			23	1			24		1		23	
*E. coli*	20	20				7		13		20						20				20		1		19	
*K. pneumoniae*	3	3				3				3						3				3				3	
*P. mirabilis*	1				1				1				1				1			1				1	
CTX-M, KPC	4	4				3		1		4				2	1	1		2		2		2	2		
*E. coli*	2	2				1		1		2				1		1				2			2		
*K. pneumoniae*	2	2				2				2				1	1			2				2			
CTX-M, NDM	1	1				1							1	1				1							1
*E. cloacae* complex	1	1				1							1	1				1							1
NDM	2				2	2							2	2				2							2
*P. aeruginosa*	2				2	2							2	2				2							2
None	69	6		58	5	5		59	5	1	58	1	9	2		63	4	2		64	3		1	58	10
*A. baumannii* complex	2	2				2							2	2				2							2
*E. cloacae* complex	1			1				1					1			1				1					1
*E. coli*	42	3		39		2		40		1	41					42				42				42	
*K. aerogenes*	1			1				1					1				1				1				1
*K. oxytoca*	2			2				2			2					2					2				2
*K. pneumoniae*	16	1		15		1		15			15	1				16				16			1	15	
*P. aeruginosa*	4				4				4				4			2	2			4					4
*P. vulgaris*	1				1				1				1				1			1				1	
Total	100	34	0	58	8	21	0	73	6	28	58	1	13	7	1	87	5	7	0	90	3	3	3	81	13

^*a*^ ESBL, extended-spectrum β-lactamase; R, resistant; I, intermediate; S, susceptible; NA, not available; +, positive; −, negative; Ind, indeterminate; REVEAL, VITEK REVEAL.

^*b*^ Gray shade indicates grouped categories summarizing isolates with the same resistance determinant and overall totals.

Four isolates carried the KPC gene, three of which were phenotypically resistant to ertapenem, imipenem, or meropenem. The other one isolate (*E. coli*) was susceptible to or yielded intermediate results for ertapenem, imipenem, and meropenem.

Three isolates (one *Enterobacter cloacae* complex and two *P. aeruginosa*) harbored the NDM gene. All three strains were resistant to, ceftazidime, imipenem, and meropenem.

Among the 69 isolates that were negative for all BCID2 resistance genes, 64 could be evaluated for phenotypic susceptibility. Of these, 9.4% (6/64) of resistance-gene-negative isolates were phenotypically resistant to cefotaxime, ceftazidime, or both, as determined by the REVEAL system. For carbapenems, all isolates were susceptible except for one isolate that showed an intermediate result to ertapenem.

## DISCUSSION

In this study, we evaluated the performance of the REVEAL fast AST system using positive blood culture samples, comparing it with QMAC-dRAST, VITEK2, and ETEST. The REVEAL system demonstrated high categorical and essential agreement rates with both fast and conventional AST platforms, meeting or exceeding the established performance thresholds for clinical implementation. Notably, this study provides one of the first head-to-head comparisons between REVEAL and QMAC-dRAST, two fast AST systems that represent fundamentally different technological approaches: QMAC-dRAST uses microcolony growth time-lapse imaging to infer antimicrobial susceptibility, while REVEAL detects changes in bacterial metabolism-associated VOC profiles. Both systems operate directly from positive blood culture bottles and require minimal hands-on time (<5 min), making them suitable for implementation in time-sensitive clinical workflows. However, their distinct detection principles, reporting formats, and system architectures require a comparative evaluation to inform laboratory adoption strategies.

In our study, REVEAL yielded 94.1% CA and 98.4% EA compared with QMAC-dRAST, with low VMD (1.4%) and MD (0.7%) rates. These performance metrics satisfy CLSI guideline M52 thresholds, recommending VMD and MD rates of ≤3% for acceptable AST systems (14). A similar performance was observed when comparing REVEAL with VITEK2 (CA: 94.5%, EA: 97.9%, VMD: 0.51%, and MD: 0.6%). Furthermore, 63.6% of the discrepant results were confirmed to be concordant with REVEAL when resolved using BMD reference method. Previous studies evaluating the REVEAL system reported overall CA and EA values ranging from 94.1% to 97.7% and between 96.1% and 98.0%, respectively, when compared with various reference or comparator methods. Our results are consistent with these previously published results, further supporting the reliability of the REVEAL system in clinical settings (11, 15–17).

While most antimicrobial-organism combinations demonstrated high concordance, we observed a lower CA (<90%) for ampicillin/sulbactam and aztreonam (vs QMAC-dRAST) and for ceftazidime and ciprofloxacin (vs VITEK2). In these instances, the discrepancies were primarily minor and might reflect the intrinsic variability in MIC interpretation thresholds across platforms.

The REVEAL system also demonstrated excellent concordance with ETEST for newer β-lactam/β-lactamase inhibitor combinations, with 100% categorical agreement for ceftazidime/avibactam and 98.6% for ceftolozane/tazobactam, revealing no major or very major errors. Although the number of carbapenem-resistant *Enterobacterales* and multidrug-resistant *P. aeruginosa* isolates was limited in this study, these findings suggest the reliability of the system for testing multidrug-resistant gram-negative infection treatment agents.

The REVEAL panel used in this study (full organism–drug matrix in Table S1) comprises over 140 organism–drug combinations, providing broad coverage across *Enterobacterales*, *Pseudomonas aeruginosa*, and *Acinetobacter baumannii* complex. In the present study, however, only 90 combinations were included in the comparative analysis, reflecting the species distribution of available isolates and the limitations of comparator testing. The tested subset incorporated most frontline and advanced therapeutic agents for Gram-negative bacteremia, including piperacillin/tazobactam, cefepime, meropenem, ceftazidime/avibactam, ceftolozane/tazobactam, aminoglycosides, carbapenems, fluoroquinolones, cephalosporins, aztreonam, and trimethoprim/sulfamethoxazole (18). Nevertheless, cefiderocol was absent from the panel, even though this agent remains critical for the management of multidrug-resistant Gram-negative bloodstream infections, particularly those caused by carbapenem-resistant *Acinetobacter baumannii* and *Pseudomonas aeruginosa* (18, 19). In contrast, tigecycline and minocycline were present in the studied panel design but have not yet received FDA clearance for use with the REVEAL system; their role in bloodstream infections is also limited, being mainly considered salvage or combination options in multidrug-resistant *Acinetobacter* infections (20). Consequently, these scenarios continue to rely on conventional AST and institution-specific therapeutic guidance.

The REVEAL system provided final AST results with a median TTR of 8.00 h, which was statistically significantly longer than that of QMAC-dRAST (median: 6.63 h), but notably faster than conventional methods. In comparison, VITEK 2 required additional subculture and exhibited a longer total TTR, further highlighting the relative efficiency of fast AST systems such as REVEAL or QMAC-dRAST. Although QMAC-dRAST demonstrated a shorter median TTR than REVEAL, the REVEAL features real-time results for each antimicrobial agent, thereby enabling clinicians to access susceptibility results as soon as available on the system monitor, without waiting for the completion of all antibiotics. The median time from loading to individual antimicrobial results was 5.97 h. When configured for progressive release, REVEAL can transmit verified, per-drug preliminary results to the electronic medical record in near real time. Notably, for critical agents used for multidrug-resistant organism treatment (e.g., ceftazidime/avibactam or meropenem), REVEAL reportedly delivered results even earlier in certain studies (17), potentially supporting earlier therapeutic modifications in time-sensitive clinical situations. When considering the optimization of antibiotic therapy within 24 h of a positive blood culture, the proportion of patients who received optimized antibiotic therapy was significantly higher in the post-intervention group (46% vs 26%, *P* = 0.037). The time to optimization in the rapid diagnostic testing group was shorter than that in the conventional group, 27 h vs 46 h, respectively (*P* < 0.001) (21). However, to realize this potential benefit, institutions should establish clear procedures to promptly review, verify, and act on newly available results. Although our study did not evaluate the clinical impact of progressive release, further research is warranted. Nonetheless, these findings support the feasibility of using the REVEAL system for urgent clinical decision-making.

In our study, genotype–phenotype correlation analyses revealed that in cases of CTX-M, KPC, or NDM genes positivity, high concordance could be observed with non-susceptible phenotypic AST results for the corresponding antibiotics, with some variable susceptibility to ceftazidime, as indicated in previous studies (22). However, among the AMR gene-negative cases, 8.6% of the isolates exhibited phenotypic resistance to cefotaxime, ceftazidime, or carbapenems, likely reflecting the presence of non-targeted resistance mechanisms such as AmpC-type β-lactamases, efflux pump overexpression, or porin loss (23–26). The risk of missing resistance in time-sensitive bloodstream infections underscores the need for rapid AMR genotypic methods to be complemented and confirmed by fast phenotypic AST methods such as REVEAL.

This study has several limitations. The sample set was collected from a single institution and consisted primarily of *E. coli* and *K. pneumoniae*, thereby limiting species diversity. Although the REVEAL system supports the testing of organisms such as *Enterobacter cloacae*, *Citrobacter freundii*, and *Citrobacter koseri*, these species were not represented in our cohort and therefore could not be evaluated. In addition, the number of non-*Enterobacterales* isolates was limited, restricting the ability of this study to comprehensively assess the performance of the REVEAL system for *Pseudomonas aeruginosa* and *Acinetobacter calcoaceticus* complex/group. This limitation also reflects the local epidemiology of bloodstream infections during the study period. In addition, certain FDA-cleared antimicrobial agents could not be evaluated due to comparator availability-related limitations. For example, ceftriaxone was excluded from the analysis, and meropenem and tobramycin were tested against *P. aeruginosa* using only the VITEK2 system (Table S1), limiting cross-platform comparison. Further studies involving a broader range of pathogens and resistance mechanisms are warranted to validate the REVEAL system entirely.

In conclusion, this study provides a robust clinical performance assessment of the VOC-based REVEAL fast AST system benchmarked against both QMAC-dRAST and conventional methods currently used in diagnostic laboratories. Its high agreement, real-time reporting, and practical workflow integration support its potential implementation as a routine and fast AST platform for managing gram-negative bloodstream infections.

## Data Availability

This study did not generate any high-throughput sequencing or large-scale data sets requiring deposition in public repositories. All data supporting the findings of this study are available within the manuscript and its supplementary materials.

## SUPPLEMENTAL MATERIAL

Figure S1 spectrum.01972-25-s0001.pdf Mean time to result per antimicrobial agent tested by the REVEAL system.

Tables S2 to S5 spectrum.01972-25-s0002.xlsx Results of antimicrobial susceptibility testing and comparison of ESBL classification across testing platforms.

Table S1 spectrum.01972-25-s0003.pdf Antimicrobial agents tested by REVEAL and corresponding comparator platforms by species.

ASM does not own the copyrights to Supplemental Material that may be linked to, or accessed through, an article. The authors have granted ASM a non-exclusive, world-wide license to publish the Supplemental Material files. Please contact the corresponding author directly for reuse.
