# Supplementary figures and images for: Clinical performance of the VITEK REVEAL fast antimicrobial susceptibility test within a real-world workflow for gram-negative bacteremia: comparison with QMAC-dRAST and conventional methods
Source: Microbiol Spectr. 2025 Nov 28;14(1):e01972-25. doi: 10.1128/spectrum.01972-25 (PMC12772308; doi:10.1128/spectrum.01972-25)

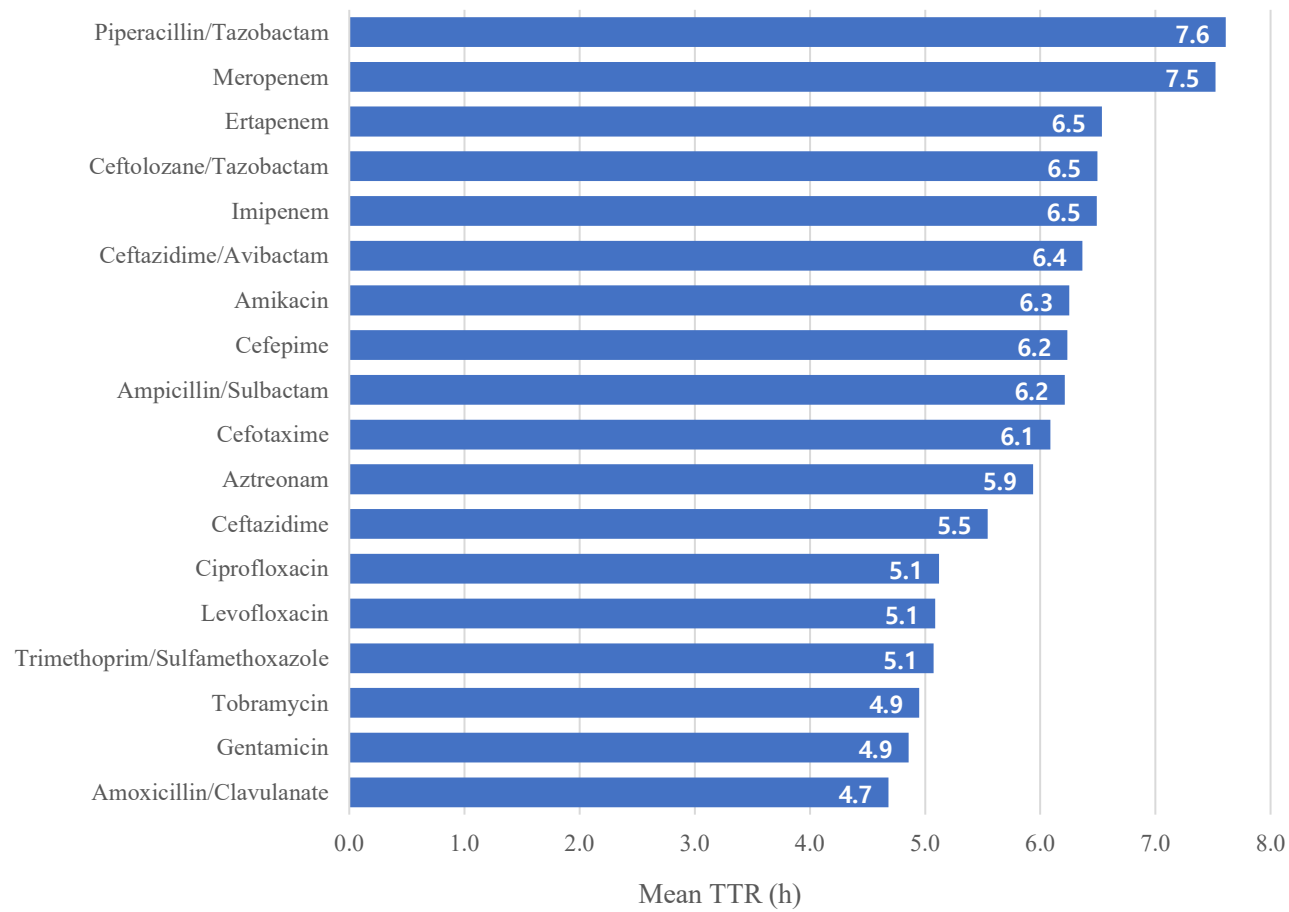

Supplementary figure 1. Mean time to result per antimicrobial agent tested by the REVEAL system

Supplement: Figure S1 — Mean time to result per antimicrobial agent tested by the REVEAL system. [file spectrum.01972-25-s0001.pdf]
