# Supplementary material for: Clinical performance of the VITEK REVEAL fast antimicrobial susceptibility test within a real-world workflow for gram-negative bacteremia: comparison with QMAC-dRAST and conventional methods
Source: Microbiol Spectr. 2025 Nov 28;14(1):e01972-25. doi: 10.1128/spectrum.01972-25 (PMC12772308; doi:10.1128/spectrum.01972-25)
Supplement: Table S1 — Antimicrobial agents tested by REVEAL and corresponding comparator platforms by species. [file spectrum.01972-25-s0003.pdf]

Supplementary table 1: Antimicrobial agents tested by REVEAL and corresponding comparator platforms by species

Antimicrobial Agents (FDA cleared)	Comparator platforms used for REVEAL evaluation across eligible species											
	E. coli	***K. pneumoniae***	***K. oxytoca***	***K. aerogenes***	***E. cloacae* complex**	***C. freundii* complex**	***C. koseri***	***P. mirabilis***	***P. vulgaris***	***S. marcescens***	***P. aeruginosa***	***A. baumannii-calcoaceticus* complex/group**
Isolates tested in this study (No.)	**64**	**21**	**2**	**1**	**2**	**0**	**0**	**1**	**1**	**0**	**6**	**2**
Amikacin	dRAST	dRAST	dRAST	dRAST	dRAST	NA		dRAST		NA	NA	dRAST‡‡
Amoxicillin/Clavulanate	dRAST/VITEK2*	dRAST/VITEK2	dRAST/VITEK2					dRAST/VITEK2††				
Ampicillin/Sulbactam	dRAST		dRAST					dRAST				
Aztreonam	dRAST	dRAST	dRAST		dRAST	NA					dRAST	
Cefepime	dRAST/VITEK2	dRAST/VITEK2	dRAST/VITEK2	dRAST/VITEK2	dRAST/VITEK2		NA				dRAST	
Cefotaxime	dRAST/VITEK2	dRAST/VITEK2	dRAST/VITEK2	dRAST/VITEK2	dRAST/VITEK2							dRAST/VITEK2
Ceftazidime	dRAST/VITEK2	dRAST/VITEK2	dRAST/VITEK2	dRAST/VITEK2	dRAST/VITEK2		NA					dRAST/VITEK2
Ceftazidime/Avibactam	ETEST	ETEST		ETEST	ETEST	NA	NA	ETEST			ETEST	
Ceftolozane/Tazobactam	ETEST		ETEST	ETEST**	ETEST		NA	ETEST	NA		ETEST	
Ceftriaxone	NA	NA	NA	NA	NA			NA				
Ciprofloxacin	dRAST/VITEK2	dRAST/VITEK2	dRAST/VITEK2	dRAST/VITEK2	dRAST/VITEK2	NA			dRAST/VITEK2	NA	dRAST/VITEK2	
Ertapenem	dRAST/VITEK2†	dRAST/VITEK2¶						dRAST/VITEK2	dRAST/VITEK2			
ESBL confirmation	dRAST/VITEK2	dRAST/VITEK2	dRAST/VITEK2									
Gentamicin	dRAST/VITEK2‡	dRAST/VITEK2	dRAST/VITEK2	dRAST		NA	NA	dRAST/VITEK2	dRAST	NA		
Imipenem	dRAST/VITEK2	dRAST/VITEK2	dRAST/VITEK2		dRAST/VITEK2		NA			NA	dRAST/VITEK2§§	dRAST/VITEK2
Levofloxacin	NA	NA	NA	NA	NA	NA	NA	NA	NA	NA	VITEK2	
Meropenem	dRAST	dRAST			dRAST			dRAST	dRAST	NA	dRAST/VITEK2	dRAST/VITEK2
Meropenem/Vaborbactam	NA	NA	NA	NA	NA	NA	NA	NA				
Piperacillin/Tazobactam	dRAST/VITEK2§	dRAST/VITEK2					NA		dRAST/VITEK2			
Tobramycin	NA	NA	NA	NA	NA	NA	NA	NA		NA	VITEK2	
Trimethoprim/Sulfamethoxazole	dRAST/VITEK2‖	dRAST/VITEK2		dRAST/VITEK2								

NA, Not available; although the antimicrobial agent and species were eligible for testing, comparative analysis was not feasible due to the unavailability of comparator testing or an insufficient number of isolates.

Gray-shaded: Not included in the analysis of this study (species not covered under REVEAL FDA-cleared claims).

dRAST, QMAC-dRAST; REVEAL, VITEK REVEAL

Note:
* REVEAL limitation: *E. coli* when the MIC is 16 μg/mL
† VITEK®2 N413 limitation: isolates with MICs of 0.25 μg/mL or 0.5 μg/mL
‡ REVEAL limitation: *E. coli* when MIC = 4 μg/mL
§ REVEAL limitation: *E. coli* with an MIC of 8 μg/mL
‖ REVEAL limitation: *E. coli* with MICs ranging from 4 to 64 μg/mL
¶ VITEK®2 N413 limitation: isolates with MICs of 0.25 μg/mL or 0.5 μg/mL
** ETEST limitation: MIC = 1 μg/mL (confirmed)
†† Limitation: *Proteus* spp., interpreted as intermediate (I) or resistant (R)
‡‡ REVEAL limitation: *A. baumannii* when MIC = 32 μg/mL
§§ REVEAL limitation: *P. aeruginosa* with an MIC of 2 μg/mL
